# Jing Si Herbal Tea Aqueous Extract Modulates G‐CSF‐Induced Hematopoietic Stem and Progenitor Cells Mobilization and Mitigates Associated Pain in a Murine Model

**DOI:** 10.1002/cbf.70335

**Published:** 2026-11-02

**Authors:** Ji‐Yan Lyu, Bruce Chi‐Kang Tsai, Chih‐Ying Chi, Yu‐Ling Wu, Wen‐Tsan Weng, Kai‐Hung Wang, Kuo‐Liang Yang, Chih‐Yang Huang, Shinn‐Zong Lin

**Affiliations:** ^1^ Institute of Medical Sciences, Tzu Chi University Hualien Taiwan; ^2^ Department of Nursing Hualien Tzu Chi Hospital, Buddhist Tzu Chi Medical Foundation Hualien Taiwan; ^3^ Department of Precision Chinese Herbal Medicine and Molecular Medicine Research E‐Da Hospital, I‐Shou University Kaohsiung Taiwan; ^4^ Department of Precision Chinese Herbal Medicine and Molecular Medicine Research E‐Da Cancer Hospital, I‐Shou University Kaohsiung Taiwan; ^5^ School of Chinese Medicine for Post‐Baccalaureate, I‐Shou University Kaohsiung Taiwan; ^6^ Department of Medical Research Hualien Tzu Chi Hospital, Buddhist Tzu Chi Medical Foundation Hualien Taiwan; ^7^ Laboratory of Immunogenetics Tzu Chi Cord Blood Bank, and Buddhist Tzu Chi Bone Marrow Donor Registry, Buddhist Tzu Chi Stem Cells Centre, Hualien Tzu Chi Hospital, Buddhist Tzu Chi Medical Foundation Hualien Taiwan; ^8^ Department of Medical Research China Medical University Hospital, China Medical University Taichung Taiwan; ^9^ Office of Research & Development Asia University Taichung Taiwan; ^10^ Superintendent's Office, Hualien Tzu Chi Hospital Buddhist Tzu Chi Medical Foundation Hualien Taiwan; ^11^ Department of Nutritional Science Fu Jen Catholic University New Taipei City Taiwan; ^12^ Bioinnovation Center, Hualien Tzu Chi Hospital Buddhist Tzu Chi Medical Foundation Hualien Taiwan; ^13^ Department of Neurosurgery Hualien Tzu Chi Hospital, Buddhist Tzu Chi Medical Foundation Hualien Taiwan; ^14^ Buddhist Tzu Chi Foundation Hospital Hualien Taiwan

**Keywords:** cannabinoid receptor 1, granulocyte colony‐stimulating factor, hematopoietic stem and progenitor cell, Jing Si Herbal Tea, pain, sodium channel Nav1.8

## Abstract

Granulocyte colony‐stimulating factor (G‐CSF) is widely used for stem cell mobilization but is associated with side effects such as pain, which often discourages healthy donors. Jing Si Herbal Tea (JSHT), a novel Chinese herbal formula comprising eight different Chinese herbal medicines, has been shown to exert pleiotropic effects against several disorders. The present study primarily explored the potential effects of JSHT aqueous extract on G‐CSF‐induced stem cell mobilization and the alleviation of G‐CSF‐induced pain. The effects of JSHT on G‐CSF‐induced hematopoietic stem and progenitor cell (HSPC) mobilization and pain‐related responses in mice were comprehensively evaluated using flow cytometric analysis, nociceptive behavioral assessments, and immunofluorescence staining. Animal studies demonstrated that JSHT had no significant effect on white blood cell (WBC) levels or HSPC mobilization. However, it helped replenish the HSPC level in the bone marrow following G‐CSF‐induced mobilization of these cells into the peripheral circulation. Furthermore, JSHT increased IFNγ expression in CD45^+^ and CD45^+^CD34^+^ cells, suggesting its potential to enhance HSPC activity. JSHT also alleviated thermal hyperalgesia, with effects comparable to those of acetaminophen (APAP), by reducing neuroinflammation and modulating the expression of cannabinoid receptor 1 (CB1) and the sodium channel Nav1.8 in the dorsal root ganglia (DRG), thereby reducing G‐CSF‐induced pain. JSHT had no effect on HSPC mobilization into the peripheral blood but might help regulate HSPC level within the bone marrow and potentially enhance HSPC function during G‐CSF treatment. Additionally, JSHT effectively attenuated G‐CSF‐induced pain, suggesting its potential as a supportive therapy during G‐CSF‐induced stem cell mobilization.

## Significance Statement

- Jing Si Herbal Tea (JSHT) addresses a critical limitation of granulocyte colony‐stimulating factor (G‐CSF) therapy—donor discomfort due to pain—without compromising hematopoietic stem and progenitor cell (HSPC) mobilization.
- This study demonstrates that JSHT preserves G‐CSF‐induced peripheral HSPC mobilization while supporting bone marrow HSPC homeostasis and potentially enhancing HSPC functional activity through IFNγ upregulation.
- Importantly, JSHT significantly alleviates G‐CSF‐induced pain by suppressing neuroinflammation and modulating CB1 and Nav1.8 signaling in the dorsal root ganglia.
- These findings identify JSHT as a promising adjunctive therapy that may improve donor compliance and comfort, offering a novel approach to optimizing stem cell mobilization protocols.

**Abbreviations**
7‐AAD: 7‐aminoactinomycin D
APAP: acetaminophen
CB1: cannabinoid receptor 1
DRG: dorsal root ganglion
GFAP: glial fibrillary acidic protein
G‐CSF: granulocyte colony‐stimulating factor
HSPCs: hematopoietic stem and progenitor cells
IFNγ: interferon γ
IL‐1β: interleukins‐1β
JSHT: Jing Si Herbal Tea
PBS: phosphate‐buffered saline
TNF‐α: tumor necrosis factor‐α
WBC: white blood cell

## 1 Introduction

Granulocyte colony‐stimulating factor (G‐CSF) is a hematopoietic growth factor that plays a key role in regulating hematopoiesis by stimulating the bone marrow to produce granulocytes and stem cells and promoting their release into the bloodstream [1, 2, 3]. Over the past decades, G‐CSF has been widely used to mobilize hematopoietic stem and progenitor cell (HSPCs) from the bone marrow into the peripheral blood, facilitating their collection for autologous or allogeneic transplantation to treat various hematological disorders, including leukemia, lymphoma, and certain genetic disorders [4, 5, 6, 7]. G‐CSF is well known for stimulating the proliferation and mobilization of hematopoietic stem cells and progenitor cells, particularly by enriching the population of cells expressing CD34 and CD45, which are surface markers of mobilized HSPCs [8, 9, 10, 11, 12, 13, 14]. Although G‐CSF is widely employed for stem cell mobilization, its administration is frequently associated with side effects, including bone pain, muscle pain, headaches, and fatigue [15, 16, 17, 18]. The common side effects of G‐CSF injections often cause discomfort among healthy peripheral blood stem cell donors and may therefore reduce their willingness to donate [17]. Healthy donors tend to experience severe pain more frequently during G‐CSF administration, with reported incidence rates ranging from 52% to 84%, which are notably higher than the rates of 25% to 38% observed in cancer patients receiving G‐CSF [19]. Therefore, reducing G‐CSF‐induced discomfort to increase the willingness of healthy individuals to donate HSPCs remains a significant challenge.

Cannabinoid receptor 1 (CB1) is a G protein‐coupled cannabinoid receptor that plays a pivotal role in mediating the effects of endocannabinoids in the peripheral and central nervous systems [20, 21, 22, 23]. The binding of endocannabinoids to CB1 inhibits presynaptic calcium channels, thereby reducing neurotransmitter release and decreasing the excitability of pain‐sensing neurons [20]. This modulation contributes to the overall analgesic effects associated with the endocannabinoid system. In addition, CB1 activation has been implicated in alleviating pain associated with inflammatory conditions by regulating neuronal excitability and inflammatory responses [24, 25, 26, 27, 28]. In addition to CB1, Nav1.8, a voltage‐gated sodium channel predominantly expressed in peripheral sensory neurons of the dorsal root ganglia (DRG), is specifically enriched in nociceptive neurons responsible for transmitting pain signals from the periphery to the central nervous system [29, 30, 31]. Nav1.8 plays a fundamental role in transducing noxious thermal and mechanical stimuli into electrical signals that are ultimately perceived as pain [30, 32, 33]. Under pathological conditions, such as inflammation or nerve injury, Nav1.8 channels are often upregulated, contributing to the hyperexcitability of nociceptive neurons [29, 30, 31, 34].

For centuries, Chinese herbal medicines and their bioactive metabolites have provided protective effects against numerous stresses and diseases, including inflammatory conditions and pain management [35, 36, 37, 38, 39]. Jing Si Herbal Tea (JSHT), an original Chinese herbal formula comprising eight different traditional Chinese herbal medicines, has been reported to exert multiple potentially beneficial biological effects [40, 41, 42, 43, 44, 45]. For instance, JSHT has been shown to effectively reduce the COVID‐19 viral load when used in combination with standard treatment [42, 46], systemic inflammation [41, 42], and lessened lung infiltrates [41, 47] in COVID‐19‐infected patients. JSHT also enhances antioxidant defenses and alleviates oxidative stress‐induced inflammation [40, 45, 47]. Furthermore, some studies have shown that the traditional Chinese herbal medicines contained in JSHT have been reported to possess anticancer and anti‐aging properties, as well as protective effects against neurological, cardiovascular, digestive, metabolic, and genitourinary disorders. Collectively, these diverse biological activities suggest that JSHT may have broad therapeutic potential for the prevention or management of various diseases. Ho et al., [42]. In addition, previous research has demonstrated that JSHT has the ability to enhance stem cell function and support their regenerative potential [43, 45]. These findings suggest that JSHT may influence the biological responses of stem and progenitor cells and thereby contribute to the maintenance of tissue homeostasis and regenerative processes.

Although previous studies have reported various potential medicinal benefits of JSHT, there is still a lack of direct evidence regarding its ability to modulate G‐CSF‐induced HSPC mobilization and pain. Therefore, in this study, we evaluated the potential of JSHT to modulate G‐CSF‐induced HSPC mobilization and alleviate the associated pain. In a G‐CSF‐mobilized mouse model, JSHT replenished the CD45^+^CD34^+^ HSPC population in the bone marrow without affecting G‐CSF‐induced mobilization and enhanced the functional activity of HSPCs mobilized by G‐CSF, as indicated by elevated IFNγ expression in both CD45^+^ and CD45^+^CD34^+^ cells in the peripheral blood and bone marrow. JSHT also significantly reduced neuroinflammatory responses in the cerebrum and spinal cord. Moreover, JSHT upregulated CB1 expression and downregulated Nav1.8 expression in the DRG, which may contribute to reduced nociceptive signaling. Taken together, our findings suggest that JSHT could support G‐CSF treatment by modulating bone marrow HSPCs, thereby enhancing their function and alleviating G‐CSF‐induced pain, while not affecting G‐CSF‐induced HSPC mobilization.

## 2 Materials and Methods

### 2.1 Chemicals Reagents

Unless stated otherwise, all the chemical reagents used in this study were of analytical grade and were obtained from Sigma‐Aldrich (St. Louis, MO, USA), United States Biological (Salem, MA, USA), or Merck (Darmstadt, Germany).

### 2.2 Animal Use and Care Ethics

The Male C57BL/6JNarl mice (Cat: RMRC11005) aged 6 weeks were purchased from the National Institutes of Applied Research National Center for Biomodels (Taipei, Taiwan). Mice were bred in the animal facility with controlled humidity, temperature, and 12 h light:12 h dark cycle with free access to food and water. All mice were allowed to acclimate for 2 weeks before further experiment. Animal use protocols were conducted according to the National Institute of Health Guide for the Care and Use of Laboratory Animals and approved (approval ID 113‐35) by The Institutional Animal Care and Use Committee (IACUC) of Hualien Tzu Chi Hospital, Hualien, Taiwan. The sample size (*n* = 5–8 per group) was determined by power analysis concerning the 3 R principle of animal ethics.

### 2.3 Preparation of Jing Si Herbal Tea (JSHT) Aqueous Extract

The preparation of JSHT was performed as previously described [45, 46]. Briefly, JSHT was a novel Chinese herbal formula prepared by the Department of Chinese Medicine at Hualien Tzu Chi Hospital, Buddhist Tzu Chi Medical Foundation, Hualien, Taiwan. A total of eight different Chinese herbal medicines were utilized in the JSHT formulation, including the following: the leaves of *Artemisiae argyi* Folium (6 g; Ai Ye; Family: Asteraceae; *Artemisia argyi* Levl. et Vant.), the leaves of *Anisomeles indica* (6 g; Yu Chen Tsao; Family: Lamiaceae; *Anisomeles indica* (L.) Kuntze.), the roots of *Platycodonis Radix* (4 g; Jie Geng; Family: Campanulaceae; *Platycodon grandiflorus* (Jacq.) A. DC.), the roots of *Ophiopogonis Radix* (4 g; Mai Men Dong; Family: Asparagaceae, *Ophiopogon japonicus (*Thunb.) Ker‐Gawl.), the whole parts of *Houttuyniae Herba* (4 g; Yu Xing Cao; Family: Saururaceae; *Houttuynia cordata* Thunb.), the leaves of *Perillae Folium* (2 g; Zi Su Ye; Family: Lamiaceae; *Perilla frutescens* (L.) Britton), the roots of *Glycyrrhiza uralensis* (2 g; Gan Cao; Family: Fabaceae; *Glycyrrhiza uralensis* Fisch. ex DC.), and the flowers of *Chrysanthemum morifolium* (0.2 g; Ju Hua; Family: Asteraceae; *Chrysanthemum × morifolium* (Ramat.) Hemsl.). The composition and proportion of the formulas were derived from the clinical experience of Chinese medicine physicians in the Department of Chinese Medicine at Hualien Tzu Chi Hospital. All Chinese herbal medicines used in JSHT were purchased by the Department of Chinese Medicine at Hualien Tzu Chi Hospital from Good Manufacturing Practice (GMP)‐certified Traditional Chinese Medicine factories approved by the Ministry of Health and Welfare (Taipei, Taiwan) and the product information of these Chinese herbal medicines including batch number, date of production/best by information product and regulatory status were recorded by the Department of Chinese Medicine at Hualien Tzu Chi Hospital. All procured Chinese herbal medicines were re‐identified by Prof. Tsung‐Jung Ho (MD, PhD, MPH) and registered Chinese medicine practitioners from the Department of Chinese Medicine at Hualien Tzu Chi Hospital. Morphological identifications were initially conducted, after which the Chinese herbal medicines were cross‐referenced with the Taiwan Herbal Pharmacopeia 4th Edition (2022, English version; available at: https://www.mohw.gov.tw/lp-3690-2.html) and the database of The Illustration of Common Medicinal Plants (Chinese version; available at: https://kmweb.moa.gov.tw/subject/subject.php?id=4595) for confirmation. All procedures involving the collection and processing of Chinese herbal medicines were conducted in full compliance with the Nagoya Protocol, CITES, relevant phytosanitary regulations, and the applicable statutes issued by the Ministry of Health and Welfare, Taipei, Taiwan. All Chinese herbal medicines were finely powdered and boiled with 600 mL of water and condensed into a 60 mL aqueous extract. The aqueous extract of JSHT was filtered with 0.45 μm filter (Cat: 295‐4545, Thermo Scientific™, Waltham, MA, USA) twice. The weight of the contents in 1 mL of the aqueous extract was determined after filtering it using a vacuum freeze dryer. The identification and characterization of the major bioactive metabolites in JSHT aqueous extract were conducted using HPLC‐MS/MS (**Supporting Material**) by Protech Technology Enterprise Co., Ltd. (Taipei, Taiwan), and the similar results have been previously published in our earlier work.

### 2.4 JSHT Treatment and G‐CSF‐Induced Mobilization in Mice

Seven experimental groups were established, as illustrated in Figure 2A, the 8‐week‐old male C57BL/6JNarl mice were randomly assigned to these seven experimental groups (*n* = 5–8 per group) as follows:

- Group 1: saline only (control group),
- Group 2: JSHT only (100 mg/kg/day),
- Group 3: G‐CSF only (250 µg/kg/day, Filgrastim M300, Kyowa Kirin Co., Ltd. Takasaki plant),
- Group 4: G‐CSF (250 µg/kg/day) + JSHT (100 mg/kg/day),
- Group 5: Acetaminophen (APAP) only (200 mg/kg/day; Cat: sc‐203425A, Santa Cruz Biotechnology, Dallas, TX, USA),
- Group 6: G‐CSF (250 µg/kg/day) + APAP (200 mg/kg/day)
- Group 7: G‐CSF (250 µg/kg/day) + JSHT (100 mg/kg/day) + APAP (200 mg/kg/day).

Mice assigned to groups receiving JSHT (Groups 2, 4, and 7) were administered JSHT at a dose of 100 mg/kg via oral gavage once daily for 15 consecutive days. Mice in the remaining groups (Groups 1, 3, 5, and 6), which did not receive JSHT, were administered an equivalent volume of saline via oral gavage following the same schedule. Beginning on day 11 of the experimental period, mice in the G‐CSF treatment groups (Groups 3, 4, 6, and 7) received subcutaneous injections of human G‐CSF (Filgrastim M300; Kyowa Kirin Co. Ltd. Takasaki Plant, Gunma, Japan) at a dose of 250 µg/kg once daily for 5 consecutive days. Mice in groups not receiving G‐CSF (Groups 1, 2, and 5) were administered an equivalent volume of normal saline subcutaneously on the same schedule. On day 12, APAP treatment was initiated in designated groups. Specifically, mice in Groups 5 and 7 received APAP at a dose of 200 mg/kg via oral gavage once daily for 4 consecutive days and mice in other groups were administered an equivalent volume of saline via oral gavage following the same schedule. The saline‐only group (Group 1) served as the normal control and G‐CSF + APAP group (Group 6) served as a positive control for analgesic treatment. At 4 h after the last G‐CSF injection on day 15, mice were anesthetized with Isoflurane (5%, Attane, Panion & BF Biotech Inc., Taipei, Taiwan), with the animal positioned on its back, the thoracic cavity was further opened, and the peripheral blood was harvested from the left ventricle of the heart with BD 3 mL Luer‐Lok syringe (Cat: 309657, Becton, Dickinson and Company, Franklin Lakes, NJ, USA) with a BD PrecisionGlide 26 G x 1/2” hypodermic needle (Cat: 305111, Becton, Dickinson and Company) and immediately transferred to the tubes containing K2EDTA anticoagulants (BD Vacutainer K2EDTA Blood Collection Tubes, Cat: 367856, Becton, Dickinson and Company). The peripheral blood was further analyzed for cell counting and flow cytometry. Bone marrow cells were harvested from the femurs and tibias of C57BL/6JNarl mice. Surrounding muscle tissue was gently removed from the femurs and tibias. The cleaned bones were placed in ice‐cold sterile PBS. To extract the bone marrow, both ends of each bone were trimmed and the marrow cavity was flushed with 5 mL of cold PBS using a 25 G needle (Cat: 305122, Becton, Dickinson and Company) connected to a 10 mL syringe (Cat: 302995, Becton, Dickinson and Company). The resulting bone marrow suspension was collected in 15 mL conical tubes, passed through a 70 μm Corning cell strainer (Cat: CLS431751, Merck) to eliminate bone fragments and debris, and centrifuged at 300 × g for 5 min at 4°C. The cell pellet was resuspended in PBS for further analysis. Besides, the organs including the brain, the L4‐L6 segments of the spinal cord, the right lumbar L3–L5 DRG also separately collected from all C57BL/6JNarl mice for further analysis.

### 2.5 Nociceptive Behavior Tests

All mice from different groups were assessed for thermal pain sensitivity on Days 13 and 14, respectively, following the completion of daily treatments (Figure 2A). All animal experiments were performed when the mice were stable and not sleeping or grooming. Thermal pain impressibility was analyzed by observing the latency of responses with the applications of thermal stimulation from a Hot Plate Analgesia Meter (Panlab) (Product Code: LE7406, Harvard Apparatus, Holliston, MA, USA). Briefly, mice were acclimated to the testing platform for 30 min prior to thermal nociceptive assessment. Thermal sensitivity was evaluated using a heated plate, and the latency to a nocifensive response was automatically recorded as an indicator of pain sensitivity.

### 2.6 Frozen Section and Fluorescent Imaging

The protocols for immunofluorescence staining of tissue were performed, as described previously [48, 49, 50]. Briefly, the fresh organs including the brain, the L4‐L6 segments of the spinal cord, and the right lumbar L3–L5 DRG, collected from C57BL/6JNarl mice on Day 15 were dissected and fixed with 4% paraformaldehyde (Cat: 158127, Sigma‐Aldrich), subjected to sucrose dehydration, and then embedded in Tissue‐Tek O.C.T. compound (Cat: PB17‐003, Sakura Finetek, Torrance, CA, USA) following by cryosections. Sections were freshly cut for 10 µM thickness in the Leica CM3050 S cryostat (Leica Biosystems, Nussloch, Germany), thaw‐mounted onto slides, and stained for immunofluorescence. The frozen sections were thawed for 15 min at room temperature and then permeabilized with 0.1% Triton‐X 100 in PBS for 10 min, blocked with 5% goat serum for 1 h, and than incubated with primary antibody separately overnight at 4°C. The primary antibodies were included with polyclonal rabbit anti‐Iba‐1 antibody (1:500 dilution; Cat: 019‐19741, FUJIFILM Wako Pure Chemical Corporation, Osaka, Japan) for the brain, monoclonal mouse anti‐glial fibrillary acidic protein (GFAP) antibody (1:1000 dilution; Cat: NBP1‐05197, Bio‐Techne, Minneapolis, MN, USA), polyclonal rabbit anti‐interleukins‐1β (IL‐1β) antibody (1:100 dilution; Cat: A20527, Abclonal, Woburn, MA, USA), polyclonal rabbit anti‐Iba‐1 antibody (1:500 dilution; Cat: 019‐19741, FUJIFILM Wako Pure Chemical Corporation) and monoclonal mouse anti‐tumor necrosis factor‐α (TNF‐α) antibody (1:50 dilution; Cat: sc‐52746, Santa Cruz Biotechnology, Dallas, TX, USA) for the spinal cord, and polyclonal rabbit anti‐Nav 1.8 antibody (1:200 dilution; Cat: ASC‐016, Alomone Lab, Jerusalem, Israel) and monoclonal rabbit anti‐CB1 antibody (1:100 dilution; Cat: ab259323, Abcam, Cambridge Biomedical Campus, Cambridge, UK) for the DRG. After washing with PBS, the sections were stained with the corresponding secondary antibodies (Alexa Fluor 488‐ or/and 594‐conjugated secondary antibodies, 1:1000 dilution; Invitrogen) followed coverslipped with ProLong Gold Antifade Mountant with DNA Stain DAPI (Cat: P36931, Invitrogen). Immunofluorescence images of tissue sections were individually captured with the OLYMPUS BX53 microscope (Olympus Corporation, Tokyo, Japan) with fluorescent photography system. The number of Iba‐1^+^ cells and the fluorescence intensity of TNF‐α, GFAP, IL‐1β, Nav1.8, and CB1 were separately analyzed by ImageJ (National Institutes of Health, Bethesda, MD, USA).

### 2.7 Detection of White Blood Cell (WBC) in Mice

After collecting mouse peripheral blood from the heart on Day 15, the WBC count was calculated using an automated hematology analyzer ((Exigo, Boule Medical AB, Stockholm, Sweden) by the Cole Parmer Microcaps Disposable Glass Micropipettes Precision‐bore glass‐capillary tube is topped with a dispensing bulb. (Cat: Cole Parmer 07950‐00, Cole‐Parmer Instrument Company, Vernon Hills, IL, USA).

### 2.8 FACS Analysis for Intracellular Cytokine and Surface Marker Expression

For HSPC analysis, the peripheral blood and bone marrow were separately harvested from C57BL/6JNarl mice. Erythrocytes in the peripheral blood were lysed using an erythrocyte‐lysis buffer (NH_4_Cl: 8.4 g/L; KHCO_3_: 1 g/L, pH = 7.4 ± 0.2, The formula was provided by LUMC Pharmacy, Leiden, The Netherlands) for further analysis. The cells in the bone marrow were harvested as described previously. Next, cells collected from the peripheral blood and bone marrow were stained with Alexa Fluor 488 anti‐mouse CD45 monoclonal antibody (Cat: 103122, Clone: 30‐F11, BioLegend) and APC anti‐mouse CD34 monoclonal antibody (Cat: 152216, Clone: SA376A4, BioLegend) in the presence of 7‐AAD (Cat: A1310, Thermo Scientific). Following wash, cells were fixed and then permeabilized with Cyto‐Fast Fix/Perm Buffer Set (Cat: 426803, BioLegend) followed by intracellular staining with PE/Cyanine7‐conjugated anti‐mouse IFNγ monoclonal antibody (Cat: 505826, Clone: XMG1.2, BioLegend) or PE/Cyanine7‐conjugated anti‐mouse TNF‐α monoclonal antibody (Cat: 506324, Clone MP6‐XT22, BioLegend). The extracellular expression of CD34^+^ in CD45^+^ cells and the intracellular expression of IFNγ or TNF‐α in CD45^+^ and CD45^+^CD34^+^ cells were then determined by BD FACSLyric™ Flow Cytometry System (BD Biosciences).

### 2.9 Statistical Analysis

All quantitative data are presented as the mean ± standard error of the mean (SEM). A one‐way analysis of variance (ANOVA) was conducted to assess statistical differences among three or more groups. When ANOVA revealed significant differences, a Tukey's post hoc multiple comparisons test was applied to identify specific group differences. Each experiment was performed with at least three independent biological replicates. All statistical analyses were performed using GraphPad Prism software (version 8.3.0 for Windows, Dotmatics, Boston, MA, USA). A *p* value less than 0.05 was considered statistically significant [51, 52].

## 3 Results

### 3.1 JSHT Modulated HSPC Mobilization in Bone Marrow Induced by G‐CSF In an In Vivo Model

A mouse model of G‐CSF‐induced HSPC mobilization was established based on a previous study [53] to investigate the effect of JSHT on G‐CSF‐induced HSPC mobilization in vivo (Figure 1A). Peripheral blood and bone marrow samples were collected from mice 4 h after the final G‐CSF injection. WBC counts and HSPCs were assessed using hematological analysis and flow cytometry, respectively. The initial results demonstrated that G‐CSF treatment alone markedly elevated the WBC count in the peripheral blood of mice (Figure 1B). However, administration of JSHT alone had no significant effect on WBC levels in the peripheral blood or on the G‐CSF‐induced increase in WBC counts (Figure 1B). Next, the percentage of CD45^+^CD34^+^ cells, a marker of HSPCs abundance, in the peripheral blood and bone marrow was analyzed using flow cytometry. Figure 2A depicts the flow cytometry gating strategies for the peripheral blood and bone marrow. The results of the HSPCs mobilization study indicated that G‐CSF increased the proportion of CD45^+^CD34^+^ cells in the peripheral blood (Figure 2B) while notably decreasing their proportion in the bone marrow (Figure 2C). These findings suggest that G‐CSF successfully mobilized HSPCs from the bone marrow niche into the peripheral blood in our mouse model. Furthermore, JSHT had no discernible impact on the proportion of CD45^+^CD34^+^ cells in the peripheral blood (Figure 2B). However, it is noteworthy that the proportion of CD45^+^CD34^+^ cells in the bone marrow increased when JSHT was administered concurrently with G‐CSF (Figure 2C). Cells isolated from the bone marrow are shown in Figure 2D. The color of the mouse bone marrow extract after G‐CSF treatment was noticeably lighter than that of the other groups. These data suggest that G‐CSF treatment significantly increased WBC counts in the peripheral blood and mobilized HSPCs from the bone marrow into the bloodstream in mice. While JSHT had no apparent effect on WBC levels or HSPC mobilization, it might help replenish HSPCs in the bone marrow that are mobilized into the peripheral blood in response to G‐CSF stimulation.

**Figure 1:**
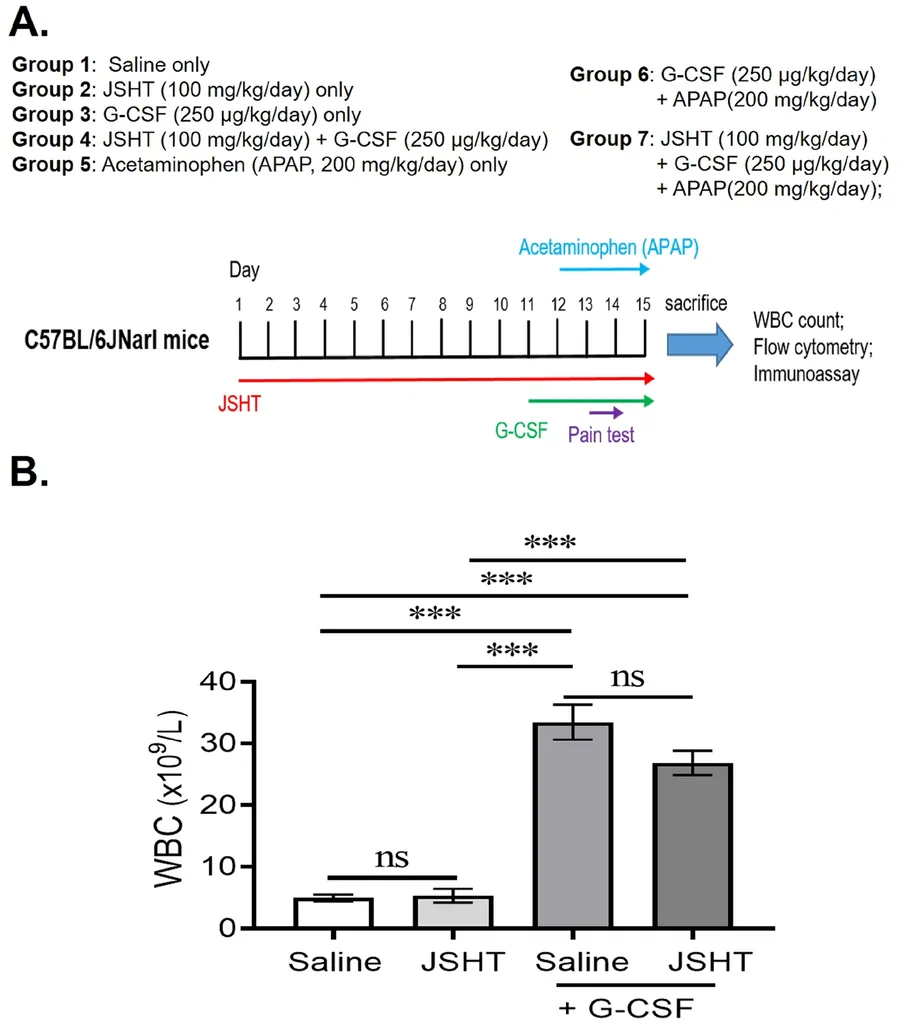
JSHT has no impact on G‐CSF‐induced elevation of peripheral white blood cells in mice. (A) The diagram outlined the animal experimental design: To assess thermal and mechanical pain sensitivities, nociceptive withdrawal latency and force were measured on Days 13 and 14. (B) G‐CSF induction significantly increased the white blood cell (WBC) count in peripheral blood, while JSHT administration had no notable impact on general WBC level and the G‐CSF‐induced elevation of WBC. Normal saline‐treated mice served as negative control. *n* = 5–8 per group. Data were expressed as mean ± SEM. ns, no significant. ***p < 0.001.

**Figure 2:**
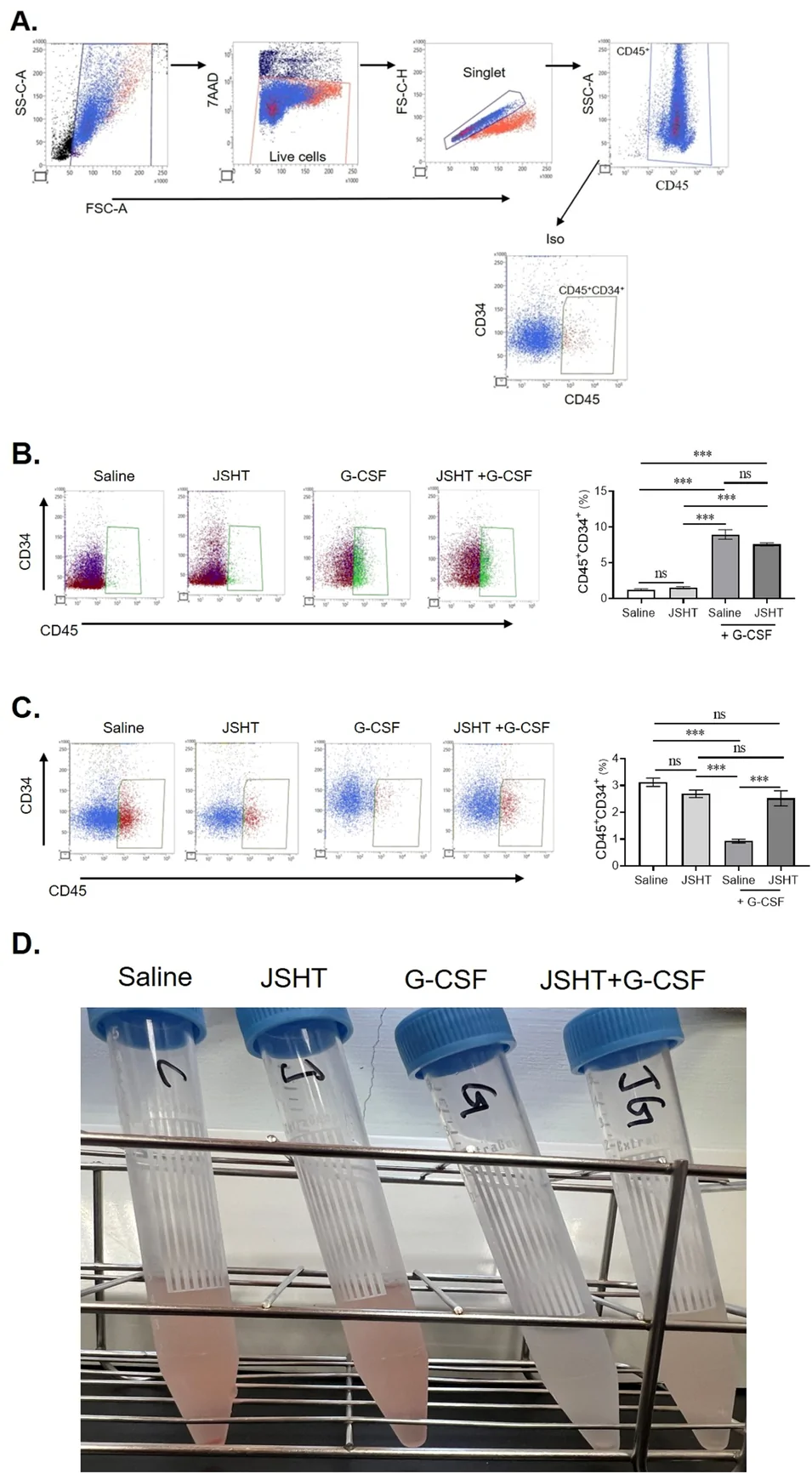
JSHT modulates G‐CSF‐induced mobilization of bone marrow hematopoietic stem and progenitor cells in vivo. (A) Gating strategy of flow cytometry analysis for the frequency of CD45 and CD34 in HSPCs in vivo. (B) The administration of G‐CSF was found to significantly increase the proportion of CD45^+^CD34^+^ cells in peripheral blood, thereby indicating the successful mobilization of HSPCs from the bone marrow. Furthermore, administration of JSHT was observed to have no significant effect on the proportion of CD45^+^CD34^+^ cells in peripheral blood. (C) The induction of G‐CSF resulted in a reduction in the existence of CD45^+^CD34^+^ cells in the bone marrow. However, the administration of JSHT with G‐CSF led to an increase in the proportion of CD45^+^CD34^+^ cells in the bone marrow. Normal saline‐treated mice served as negative control. (D) The bone marrow extract from mice treated with G‐CSF appeared significantly lighter in color compared to the other groups. *n* = 5–8 per group. Data were expressed as mean ± SEM. ns: no significant. ***p < 0.001.

### 3.2 JSHT Enhanced IFNγ Expression in HSPCs of Peripheral Blood and Bone Marrow Following G‐CSF‐Induced Mobilization

Prior studies have demonstrated that stem cell mobilization and homeostasis are associated with higher levels of proinflammatory cytokines, such as IFNγ and TNF‐α [54, 55, 56, 57, 58, 59]. To further investigate this effect, the expression of proinflammatory cytokines, including IFNγ and TNF‐α, in HSPCs from the peripheral blood and bone marrow was analyzed. The flow cytometry gating strategies for the peripheral blood and bone marrow are presented in Figure 3A. G‐CSF treatment reduced IFNγ expression within the CD45^+^ cell population in both the peripheral blood (Figure 3B) and bone marrow (Figure 3C). However, JSHT administration altered the G‐CSF‐induced reduction in IFNγ expression within the CD45^+^ cell population in both the peripheral blood (Figure 3B) and bone marrow (Figure 3C), with a more pronounced effect in the peripheral blood. Similarly, JSHT increased IFNγ expression within the CD45^+^CD34^+^ cell population, which had been reduced by G‐CSF stimulation, in both the peripheral blood (Figure 4A) and bone marrow (Figure 4B), particularly in the peripheral blood. The increased IFN‐γ expression observed within the CD45^+^CD34^+^ HSPC‐enriched population suggests that JSHT may modulate the inflammatory or activation state of hematopoietic stem and progenitor cells during G‐CSF‐induced mobilization. Additionally, the data revealed that JSHT significantly enhanced TNF‐α expression within the CD45^+^ cell population in the peripheral blood, which had been reduced by G‐CSF (Figure 4C). However, no significant differences in TNF‐α expression were observed in either the CD45^+^ (Figure 4D) or CD45^+^CD34^+^ (Figure 4E) cell populations in the bone marrow among the experimental groups. Furthermore, TNF‐α expression in the CD45^+^CD34^+^ cell population in the peripheral blood was minimal and below the detection limit under our experimental conditions. These findings suggest that JSHT may enhance the functional activity of G‐CSF‐induced HSPCs in both the peripheral blood and bone marrow, potentially through the upregulation of IFNγ expression in HSPCs.

**Figure 3:**
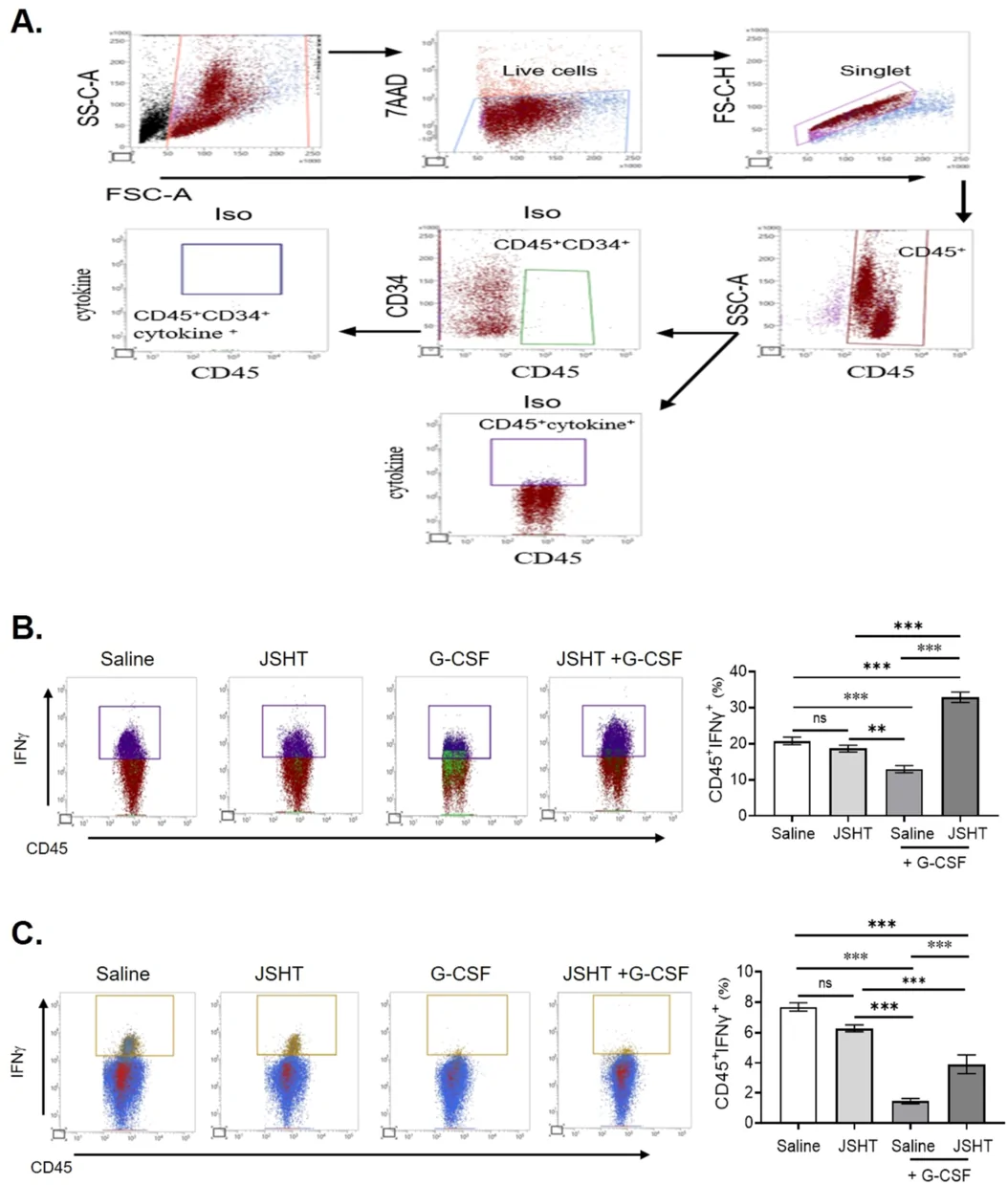
JSHT enhances IFNγ expression in CD45^+^ HSPCs of peripheral blood and bone marrow following G‐CSF‐induced mobilization. (A) Gating strategy of flow cytometry analysis for the frequency of proinflammatory cytokines in HSPCs in vivo. (B, C) Effect of G‐CSF and JSHT on IFNγ expression within the CD45^+^ cell population in peripheral blood and bone marrow. G‐CSF treatment reduced the expression of IFNγ in the CD45^+^ cell population in both peripheral blood (B) and bone marrow (C). Co‐administration of JSHT altered this G‐CSF‐induced reduction, with a more pronounced effect in the peripheral blood. Normal saline‐treated mice served as negative control. *n* = 5–8 per group. Data were expressed as mean ± SEM. ns: no significant. **p < 0.01. ***p < 0.001.

**Figure 4:**
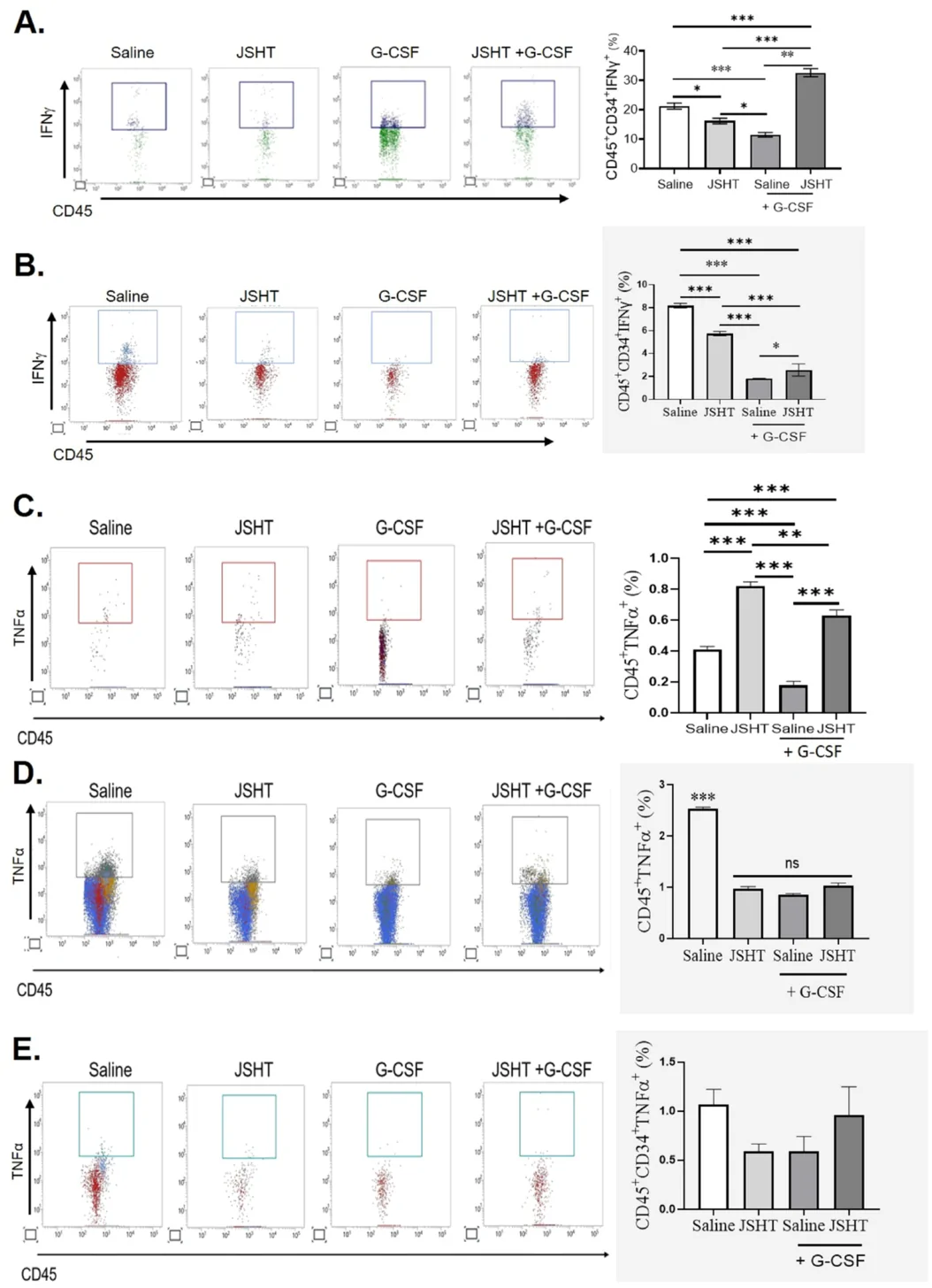
JSHT modulates the expression of IFNγ and TNF‐α in different HSPC populations in peripheral blood and bone marrow following G‐CSF induction. (A, B) JSHT increased the expression of IFNγ within the CD45^+^CD34^+^ cell population, which had been reduced by G‐CSF treatment. This effect was observed in both the peripheral blood (A) and bone marrow (B). Notably, the enhancement of IFNγ expression by JSHT was more pronounced in the peripheral blood. (C) JSHT also significantly increased the expression of TNF‐α within the CD45^+^ cell population in the peripheral blood under G‐CSF induction. (D, E) No significant changes were observed in TNF‐α expression in either the CD45^+^ cell population or the CD45^+^CD34^+^ cell population within the bone marrow of the treated mice, indicating that JSHT did no significantly alter TNF‐α level of HSPCs in the bone marrow under G‐CSF induction. Normal saline‐treated mice served as negative control. *n* = 5–8 per group. Data were expressed as mean ± SEM. ns: no significant. *p < 0.05. **p < 0.01. ***p < 0.001.

### 3.3 JSHT Ameliorated G‐CSF‐Induced Pain in Mice as Measured by Thermal Pain Behaviors

In addition, the beneficial effect of JSHT in mitigating G‐CSF‐induced pain was also evaluated, as G‐CSF treatment commonly causes headaches, musculoskeletal pain, bone pain, and visceral pain [60, 61]. Based on these findings, we evaluated thermal pain sensitivity in mice treated with G‐CSF for at least three consecutive days, on days 13 and 14. The results indicated that G‐CSF injection resulted in a notable reduction in the response threshold to thermal stimuli in mice (Figure 5). Importantly, the administration of JSHT to G‐CSF‐injected mice reduced G‐CSF‐induced hyperalgesia, and its analgesic effect was comparable to that of APAP. Interestingly, the combination of JSHT and APAP did not result in a notably enhanced analgesic effect (Figure 5). These findings suggest that JSHT may possess analgesic properties capable of alleviating G‐CSF‐induced pain.

**Figure 5:**
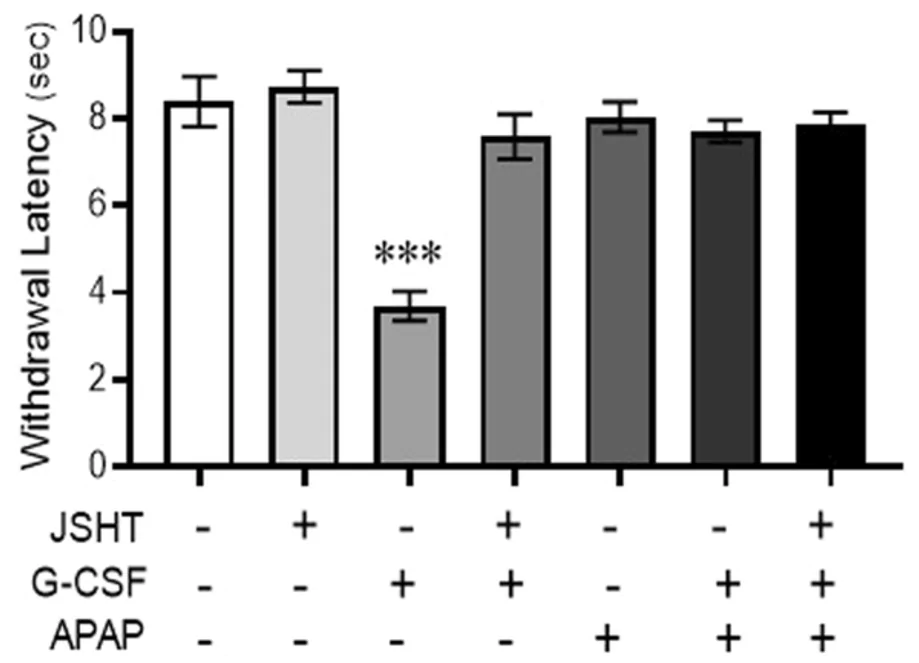
JSHT significantly ameliorates the G‐CSF‐induced pain on thermal pain behaviors. The nociceptive withdrawal latency was separately shown by the thermal pain sensitivity. Injection of G‐CSF resulted in a notable reduction in response thresholds to thermal stimulation. It is noteworthy that the administration of JSHT reduced the G‐CSF‐induced hyperalgesia and the analgesic effect was comparable to that of acetaminophen (APAP). Normal saline‐treated mice served as negative control. *n* = 5–8 per group. Data were expressed as mean ± SEM. ***p < 0.001.

### 3.4 JSHT Attenuated the G‐CSF‐Activated Inflammation in the Center Nervous System

Previous studies have indicated that G‐CSF has the ability to modulate pain perception via inflammation [62] and JSHT has been reported to exhibit anti‐inflammatory properties [40, 41, 42, 45, 46, 47]. Therefore, to investigate whether JSHT mitigated G‐CSF‐induced pain by inhibiting inflammatory responses, we examined cerebrum of brain and spinal cord tissues from mice model using immunofluorescence staining. In the brain, it was observed that G‐CSF significantly increased the number of microglial cells (Iba‐1 positive cells) in the cerebrum of brain [63, 64]. Nevertheless, the administration of JSHT was observed to markedly reduce the number of microglial cells, thereby indicating its potential capacity to attenuate G‐CSF‐induced neuroinflammation in the cerebrum of brain (Figure 6A). A similar effect was observed in the spinal cord, where G‐CSF induced the upregulation of microglial cells and inflammatory mediators, including TNF‐α, IL‐1β, and GFAP (Figure 6B,C; Figure S1). JSHT reduced the increase in these inflammatory markers induced by G‐CSF in the spinal cord (Figure 6B,C). These findings suggest that JSHT alleviates G‐CSF‐induced pain by inhibiting microglial activation in the cerebrum of brain and spinal cord as well as reducing inflammatory responses in the spinal cord.

**Figure 6:**
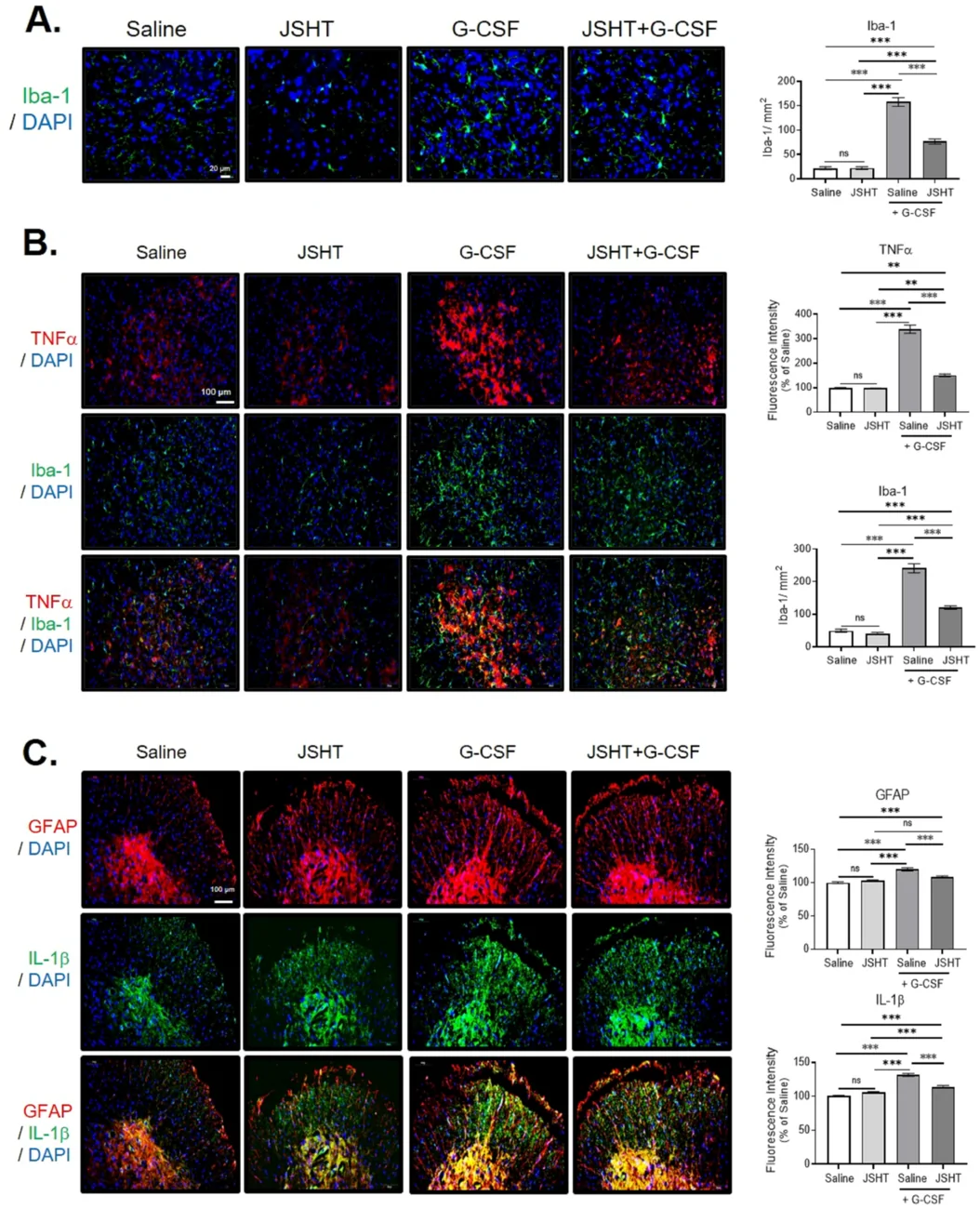
JSHT attenuates the inflammation in the central nervous system. 4 h after the G‐CSF injection on the last day, we collected the brains and spinal cords of saline‐, JSHT (100 mg/kg)‐, G‐CSF (250 µg/kg)‐, and JSHT (100 mg/kg) + G‐CSF (250 µg/kg)‐treated mice separately. The tissues were further subjected to immunofluorescent staining with Iba‐1 in the cerebrum of brain (A), Iba‐1 co‐stained with TNF‐α (B), and GFAP co‐stained with IL‐1β (C) in the spinal cord. G‐CSF induction increased microglial cells (Iba‐1 positive cells) in the cerebrum of brain and spinal cord and inflammatory markers (TNF‐α, IL‐1β, GFAP) in the spinal cord, while JSHT treatment effectively suppressed these responses. The representative images of each group were shown. The fluorescence intensity of each staining was also quantified. Scale bars, 20 µM (A) and 100 µm (B and C). Normal saline‐treated mice served as negative control. Data were expressed as mean ± SEM. ns: no significant. **p < 0.01. ***p < 0.001.

### 3.5 JSHT Promoted the Expression of CB1 and Reduced Level of Nav 1.8 in the DRG

As CB1 and Nav1.8 have been shown to modulate pain perception [20, 50, 65, 66], the next objective of this study was to determine whether changes in CB1 and Nav1.8 expression contributed to the pain relief afforded by JSHT in G‐CSF‐induced pain, as assessed by immunofluorescence staining. In the mouse DRG, G‐CSF stimulation led to an increase in Nav1.8 expression and a decrease in CB1 expression (Figure 7). A reduction in CB1/Nav1.8 colocalization was also observed in the DRG (Figure 7A). Notably, JSHT modulated these G‐CSF‐induced changes by downregulating Nav1.8 (Figure 7A,B), upregulating CB1 (Figure 7A,C), and restoring the colocalization of CB1 and Nav1.8 (Figure 7A). These findings suggest that JSHT mitigated G‐CSF‐induced pain by modulating the expression and interaction of CB1 and Nav1.8 in the DRG.

**Figure 7:**
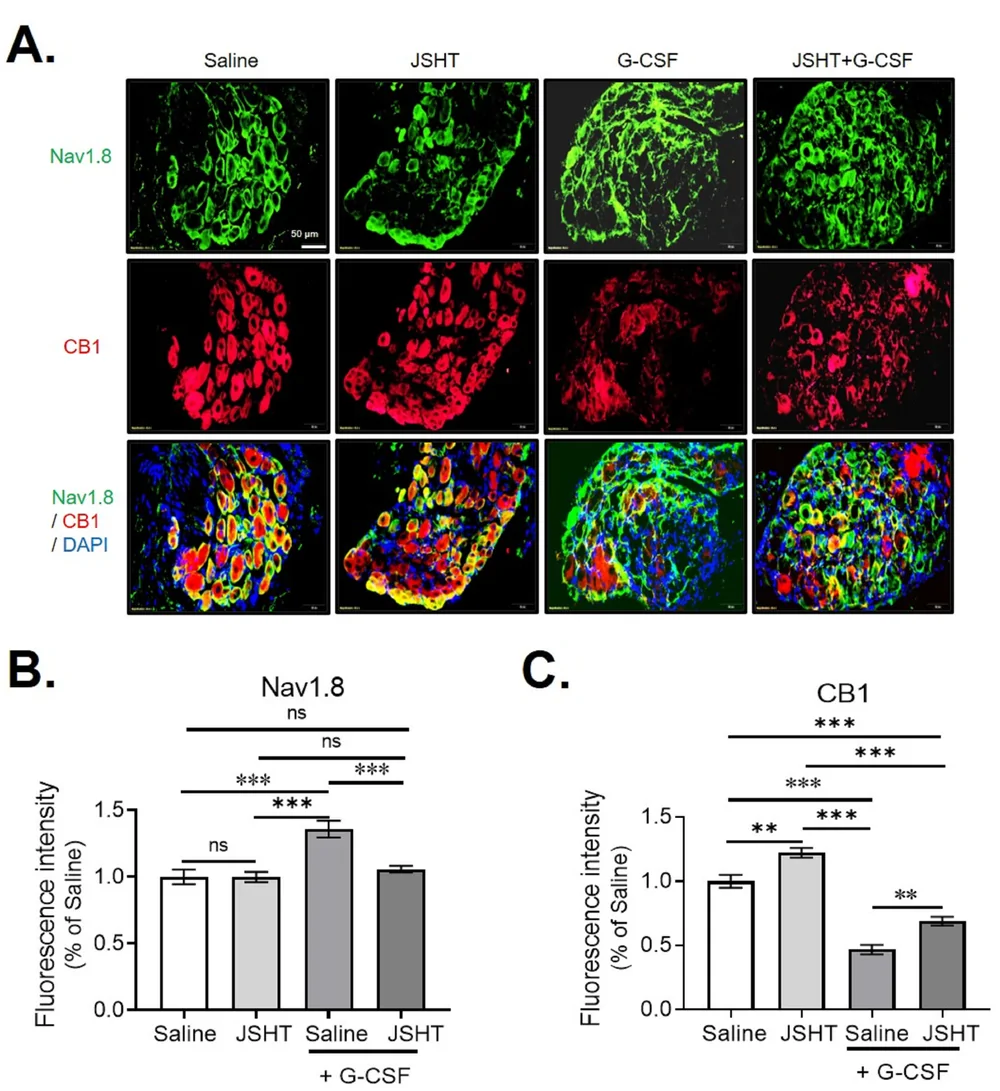
JSHT promotes expression of CB1 and reduces level of Nav 1.8 in the DRG. 4 h after the G‐CSF injection on the last day, the L3–L5 lumbar DRG of saline‐, JSHT (100 mg/kg)‐, G‐CSF (250 µg/kg)‐, and JSHT (100 mg/kg) + G‐CSF (250 µg/kg)‐treated mice were collected and subjected to immunofluorescent staining to explore Nav1.8 and CB1 expression. (A) The representative images of Nav1.8 and CB1 staining in the DRG of each group were shown. Scale bar, 50 µm. The fluorescence intensity of Nav1.8 (B) and CB1 (C) in the DRG was also quantified. G‐CSF stimulation upregulated Nav1.8 expression and downregulated CB1 expression in the DRG, whereas JSHT administration altered these changes. Normal saline‐treated mice served as negative control. Data were expressed as mean ± SEM. ns: no significant. **p < 0.01. ***p < 0.001.

## 4 Discussion

The present study investigated the effects of JSHT on G‐CSF‐induced HSPC mobilization as well as G‐CSF‐associated pain responses. (1) G‐CSF significantly induced HSPC mobilization, which was unaffected by JSHT pretreatment; however, JSHT might modulate the CD45^+^CD34^+^ HSPC population in the bone marrow during mobilization. (2) JSHT upregulated IFNγ expression in the CD45^+^ and CD45^+^CD34^+^ cell populations. (3) JSHT alleviated G‐CSF‐induced thermal hyperalgesia to a degree comparable to that of APAP. However, no significant synergistic effect was observed when JSHT and APAP were administered together. (4) JSHT reduced G‐CSF‐induced neuroinflammation in the cerebrum and spinal cord and alleviated pain by modulating the expression and colocalization of CB1 and Nav1.8 in the DRG.

G‐CSF is widely used to mobilize HSPCs from the bone marrow into the peripheral blood [67]. JSHT, a formulation composed of eight distinct Chinese herbal medicines, has been shown to exert pleiotropic effects in combating metabolic, neurological, cancer‐related, cardiac damage, and age‐associated disorders [40, 41, 42, 43, 45]. The previous study reported that the cardioprotective potential of human adipose‐derived stem cells is enhanced through JSHT treatment [43], indicating the potential benefit of JSHT on stem cell function. In the current study, JSHT treatment provided important insights into its effects on G‐CSF‐induced HSPC mobilization in both the peripheral blood and bone marrow in the in vivo model. JSHT administration had little effect on the WBC count (Figure 1) or the number of CD45^+^CD34^+^ cells in the peripheral blood following G‐CSF induction (Figure 2), but it modulated the population of CD45^+^CD34^+^ cells in the bone marrow after G‐CSF induction (Figure 2). Additionally, the mobilization and maintenance of hematopoietic stem cells are influenced by the activity of proinflammatory cytokines, such as IFNγ and TNF‐α, as indicated by previous studies [54, 55, 56, 57, 58, 59]. In the current study, our results showed that JSHT treatment primarily enhanced IFN‐γ expression in the mobilized CD45^+^ and CD45^+^CD34^+^ cell populations in both the peripheral blood and bone marrow under G‐CSF stimulation (Figure 3B,C and Figure 4A,B). However, a significant increase in TNF‐α expression, which had been reduced by G‐CSF, was observed only in the CD45^+^ cell population in the peripheral blood following JSHT treatment (Figure 4C), but not in the other cell populations (Figure 4D,E). IFN‐γ has been reported to exert dual effects on HSPCs. Short‐term exposure stimulates their proliferation, whereas prolonged signaling promotes differentiation toward myeloid effector cells and impairs their long‐term self‐renewal capacity [57, 58, 59]. Based on previous research and our findings, although JSHT may not notably increase the overall G‐CSF‐induced mobilization of HSPCs into the peripheral blood, it could potentially enhance the activity and functional capacity of the mobilized HSPCs population by regulating inflammatory cytokine expression. The bioactive metabolites in JSHT may have different effects on HSPCs mobilization and stem cell activity during G‐CSF induction. It is noteworthy that, while JSHT had no significant impact on G‐CSF‐induced HSPCs mobilization into the peripheral blood, it might produce distinct mobilization patterns in the bone marrow. G‐CSF can effectively mobilize HSPCs but disrupt HSPCs homeostasis in the bone marrow [68]. In contrast, our data suggest that JSHT supports the balanced modulation of HSPC. In the present animal model, co‐administration of JSHT with G‐CSF was mainly associated with a higher proportion of CD45^+^CD34^+^ cells in the bone marrow without impairing peripheral mobilization. This observation suggests a possible role for JSHT in supporting bone marrow HSPC homeostasis during G‐CSF treatment; however, further investigations are warranted to explore its potential clinical application.

Another important finding in this study was the analgesic efficacy of JSHT. Generally, the G‐CSF application is often associated with varying degrees of pain, including bone, muscle, and headache pain [60, 61]. Therefore, identifying effective adjunctive treatments with minimal side effects to alleviate G‐CSF‐induced pain has become a clinical research priority. This study is the first to demonstrate that JSHT alleviated G‐CSF‐induced pain, thereby normalizing pain‐related behavior in mice. Furthermore, the analgesic effect of JSHT was comparable to that of APAP (Figure 5). To investigate the analgesic mechanism of JSHT, we found that JSHT attenuated G‐CSF‐induced inflammation in the central nervous system. Previous studies have shown that G‐CSF stimulates microglia, leading to the sensitization of DRG neurons. Iba‐1 serves as both a marker and an indicator of microglial activation [63, 64, 69]. In our study, G‐CSF induction increased level of microglial cell in the cerebrum of brain (Figure 6A) and spinal cord (Figure 6B), but JSHT administration reduced this effect. Similarly, the inflammatory mediators and pain‐related factors, including TNF‐α [70], IL‐1β [71], and GFAP (a marker for activation of astrocytes) [72, 73], were elevated in the spinal cord following G‐CSF induction but were suppressed by JSHT (Figure 6B,C). These findings suggest that JSHT may alleviate neuroinflammation and related pain activation in the cerebrum of brain and spinal cord.

Moreover, the present study showed that G‐CSF induction reduced CB1 levels, whereas JSHT upregulated CB1 expression in the DRG of G‐CSF‐treated mice (Figure 7). The DRG, located between the spinal cord and peripheral nerves, is an essential site where the endocannabinoid system modulates incoming pain signals [20, 22, 31, 74]. Previous researches confirm the benefit of CB1 to mitigate cancer‐induced bone pain [75, 76]. Therefore, enhancing CB1 expression may activate endogenous analgesic mechanisms, thereby alleviating bone pain. Concurrently, we found that G‐CSF induced a significant upregulation of the voltage‐gated sodium channel Nav1.8 in the DRG; however, JSHT treatment significantly reduced its expression (Figure 7). Nav1.8 plays an essential role in transmitting peripheral noxious stimuli to the central nervous system, and its overactivation may exacerbate pain perception [29, 31, 34, 77]. These findings suggest that JSHT may exert a comprehensive analgesic effect by synergistically modulating multiple molecular pathways, including the Nav1.8 ion channel, CB1 activity, and inflammatory responses. The reduction in inflammatory responses in the cerebrum and spinal cord by JSHT could contribute to the alleviation of central sensitization. Furthermore, the upregulation of CB1 in the DRG may enhance the role of the endocannabinoid system in modulating nociceptive signaling, thereby reducing pain transmission. The downregulation of Nav1.8 by JSHT could play a pivotal role in attenuating the hyperexcitability of nociceptive neurons, which is often associated with pain perception. These findings provide new evidence supporting the use of Chinese herbal medicines to modulate G‐CSF‐induced pain.

JSHT comprises eight different Chinese herbal medicines, including *Artemisia argyi* Folium, *Anisomeles indica*, *Platycodonis Radix*, *Ophiopogonis Radix*, *Houttuyniae Herba*, *Perillae Folium*, *Glycyrrhiza uralensis*, and *Chrysanthemum morifolium*. Currently, there is no clear evidence indicating notable antagonistic interactions among the Chinese herbal medicines in JSHT. However, further investigation is necessary to determine whether any potential antagonistic interactions among these herbal medicines could reduce the therapeutic efficacy of JSHT. According to previous studies, most of the Chinese herbal medicines included in JSHT exhibit notable anti‐inflammatory properties, which may contribute to its therapeutic potential [42, 78, 79, 80, 81, 82, 83, 84]. Additionally, some ingredients in JSHT also exhibit potential analgesic properties. For instance, *Artemisia argyi* Folium has been shown to alleviate neuropathic pain [85]. The water extract from the leaves and stems of *Anisomeles indica* prior to flowering exhibits both analgesic and antihyperalgesic effects in rats [86]. *Perillae Folium* is traditionally used for pain relief, particularly in the treatment of headaches [87]. *Glycyrrhiza uralensis* holds promise as a pain‐relieving agent, although its regular use may be associated with adverse effects [88]. Moreover, Bone pain caused by G‐CSF has also been linked to the release of histamine as part of the inflammatory response in the body [16]. Previous studies have reported that that *Artemisia argyi* Folium [89], *Anisomeles indica* [90], *Platycodonis Radix* [91], *Houttuyniae Herba* [92], *Perillae Folium* [93], and *Glycyrrhiza uralensis* [94] exhibit antihistaminic properties. Together, these findings suggest that JSHT contains multiple Chinese herbal medicines with anti‐inflammatory properties. Some of these Chinese herbal medicines also contribute to pain relief through antihistaminic properties or other mechanisms that regulate pain. Therefore, it can be hypothesized that the combination of Chinese herbal medicines in JSHT may exert synergistic or additive effects, thereby enhancing its overall efficacy in pain management. However, given the complex mechanisms underlying G‐CSF‐induced pain perception [16, 19], the present study only focused primarily on G‐CSF‐activated inflammation in the central nervous system and the expression of CB1 and Nav1.8 in the DRG. In addition, although previous studies have reported the beneficial effects of JSHT and its ingredients on stem cells [43, 45, 95, 96], no prior research has demonstrated its influence on G‐CSF‐induced HSPC mobilization, either as the formulation or through its individual Chinese herbal medicines.

Moreover, the results of HPLC‐MS/MS (**Supporting Material**) shew JSHT mainly contained harmine (very small amount), swertisin, isoliquiritigenin, eupatilin, 3’,4’‐dimethoxyflavone, and nerolidol, and the similar results have been previously published in our earlier work [41, 45, 97]. Harmine, though present in low amounts in JSHT, is known to inhibit inflammation through the repression of NF‐κB signaling [98] and has been shown to reduce pain‐like behaviors in an animal model [99]. Swertisin perhaps have anti‐inflammatory properties [100] and has demonstrated potential in relieving pain [101]. Isoliquiritigenin is a well‐documented anti‐inflammatory agent [102, 103] that also alleviates neuropathic pain [104], possibly through inhibition of Nav1.8 [105]. Eupatilin has shown therapeutic efficacy in various inflammatory conditions, including intracranial hemorrhage and chronic rhinosinusitis [106, 107] and has also been reported to reduce pain and cartilage degradation in osteoarthritis models [108]. Similarly, 3’,4’‐dimethoxyflavone exhibits anti‐inflammatory and neurotrophic or neuroprotective effects that may be crucial for suppressing neuropathic pain [109]. Lastly, nerolidol has demonstrated significant anti‐inflammatory activity [110] and analgesic effects, particularly through modulation of the GABAergic system, which is known to be involved in neuropathic pain regulation [111]. Likewise, G‐CSF‐induced bone pain is thought to be associated with histamine release triggered by the inflammatory response in the body [16]. Swertisin exhibits a relatively weak inhibitory effect on hyaluronidase, an enzyme involved in mast cell‐mediated histamine release during inflammatory responses [112]. In contrast, isoliquiritigenin has been shown to suppress related mechanisms of histamine release in LAD2 mast cells and significantly reduce histamine release in a murine paw edema model [113]. Similarly, eupatilin effectively inhibits histamine release in allergic inflammation models induced by compound 48/80 [114]. However, there is currently no direct evidence confirming that harmine, 3′,4′‐dimethoxyflavone, or nerolidol can reduce histamine release. Taken together, the reported effects of these bioactive metabolites suggest that JSHT may exert protective and analgesic effects against G‐CSF‐induced pain through multifaceted mechanisms involving anti‐inflammatory activity, modulation of nociceptive pathways (e.g., Nav1.8 and the GABAergic system), reduction of histamine release, and neuroprotective effects. Moreover, among these bioactive metabolites, there is currently no scientific evidence indicating that eupatilin has notable effects on stem cells. In contrast, other bioactive metabolites have been reported to exert effects on different types of stem cells. Swertisin promotes the formation of new islets from pancreatic stem and progenitor cells under diabetic conditions [115, 116]. Isoliquiritigenin has the ability to promote the transdifferentiation of white adipocytes, derived from human adipose‐derived stem cells, into metabolically active beige and brown adipocytes [117]. Prior research has shown that when human hematopoietic stem cells derived from mobilized peripheral blood are cultured with 3′,4′‐dimethoxyflavone under normoxic conditions, CD34 positivity is highly preserved after 1 week. This treatment also resulted in a 27.8‐fold increase in the number of CD34^+^ cells, which was significantly higher than the 14.4‐fold increase observed in the control group [118]. Treatment with nerolidol effectively restored granulocyte levels and G‐CSF concentrations in cyclophosphamide‐treated groups. Nerolidol also preserved bone marrow architecture and significantly increased bone marrow cell counts [119]. Although harmine has been shown to limit endogenous G‐CSF expression in LPS‐induced acute lung injury [120], it was present in very low amounts in JSHT and there are no obvious reports that mention its ability to reduce G‐CSF‐induced HSPC mobilization. These findings suggest that some of the bioactive metabolites in JSHT may regulate stem cell proliferation and differentiation, potentially enhancing G‐CSF‐induced HSPC mobilization. However, further studies are necessary to clarify the precise mechanisms underlying these effects.

Although the results of this study are promising, several limitations must be acknowledged. First, this study was conducted exclusively in male C57BL/6JNarl mice. While this design minimized variability associated with hormonal fluctuations in females, it restricts the generalizability of our findings. Given the well‐documented sex differences in pain perception and analgesic responses, future studies should include female mice to determine whether sex‐specific effects are present. Second, only a single dose of JSHT was tested, based on previous studies in diabetic mouse models [45]. This limits our understanding of the dose–response relationship, efficacy thresholds, and potential toxicity range. Future studies should therefore employ a broader range of doses to better define the pharmacological profile and therapeutic window of JSHT. Third, although CD45^+^CD34^+^ cells were used to identify an HSPC‐enriched population, this phenotype encompasses a heterogeneous population of hematopoietic stem and progenitor cells and does not allow identification of specific definitive hematopoietic stem cells. The absence of more stringent phenotypic markers, such as Lineage^−^Sca‐1^+^c‐Kit^+^ markers and additional markers for long‐term hematopoietic stem cells, together with the lack of functional assays such as colony‐forming unit assays and long‐term repopulation studies, limits our ability to determine the specific effects of JSHT on hematopoietic stem cell self‐renewal, differentiation, and long‐term hematopoietic function. Therefore, future studies should incorporate more specific hematopoietic stem cell phenotyping and functional assays. Fourth, although JSHT enhanced cytokine expression, such as IFNγ, in hematopoietic cell populations, it remains unclear whether this reflects local cellular activity or systemic inflammation. Plasma cytokine measurements would help clarify the systemic immunomodulatory effects of JSHT during G‐CSF treatment. Another limitation is the lack of assessment of histamine levels and mast cell activation, which may contribute to G‐CSF‐induced bone pain. Moreover, our analyses focused primarily on neuroinflammation and CB1/Nav1.8 expression without fully addressing other potential pain mechanisms. Although JSHT treatment altered CB1 and Nav1.8 expression in the DRG, the causal contribution of these molecular changes to the analgesic effects of JSHT was not directly established. Future studies using selective CB1 antagonists and Nav1.8‐specific pharmacological or genetic approaches are warranted to determine whether these pathways are functionally required for the analgesic effects of JSHT. Additional experiments, including evaluation of peripheral sensitization pathways, will also be necessary to more comprehensively elucidate the analgesic mechanisms of JSHT. Furthermore, although several bioactive metabolites of JSHT were identified, their individual contributions to HSPC mobilization and analgesia remain undefined. Without detailed pharmacokinetic profiling, the bioavailability, metabolism, and potential toxicity of these compounds cannot be fully determined. Finally, although the current findings were obtained in mice, clinical studies are essential to establish the safety and efficacy of JSHT in supporting G‐CSF‐induced hematopoietic stem/progenitor cell mobilization and alleviating associated pain in humans.

## 5 Conclusion

In summary, our study demonstrated that, while JSHT had no significant effect on G‐CSF‐induced mobilization of HSPCs from the bone marrow into the peripheral blood, it might help modulate the CD45^+^CD34^+^ HSPC population in the bone marrow during mobilization. Additionally, JSHT upregulated IFNγ expression in HSPC‐related cell populations, suggesting a potential enhancement of stem cell functionality. Notably, JSHT exhibited significant analgesic effects by reducing G‐CSF‐induced hyperalgesia and neuroinflammation in the cerebrum and spinal cord. JSHT also modulated CB1 and Nav1.8 expression in the dorsal root ganglia. These findings suggest that JSHT may serve as an adjunctive therapy to alleviate G‐CSF‐induced pain and support bone marrow HSPC homeostasis during mobilization.

## Author Contributions

**Ji‐Yan Lyu:** conceptualization, formal analysis, investigation, data curation, visualization, funding acquisition, and writing – review and editing. **Bruce Chi‐Kang Tsai:** formal analysis, visualization, validation, and writing – original draft preparation. **Chih‐Ying Chi:** methodology, formal analysis, validation and writing – review and editing. **Yu‐Ling Wu:** methodology, formal analysis, validation and writing – review and editing. **Wen‐Tsan Weng:** conceptualization, methodology, formal analysis, investigation, data curation, visualization, and writing – original draft preparation. **Kai‐Hung Wang:** methodology, investigation, and writing – review and editing. **Kuo‐Liang Yang:** conceptualization, methodology, and writing – review and editing. **Chih‐Yang Huang:** resources, funding acquisition, project administration, data curation, and writing – review and editing. **Shinn‐Zong Lin:** resources, supervision, project administration, funding acquisition, and writing – review and editing. All authors have read and agreed to the published version of the manuscript.

## Ethics Statement

Animal use protocols were conducted according to the National Institute of Health Guide for the Care and Use of Laboratory Animals and approved (approval ID 113‐35) by The Institutional Animal Care and Use Committee (IACUC) of Hualien Tzu Chi Hospital, Hualien, Taiwan.

## Conflicts of Interest

The authors declare no conflicts of interest.

## Supporting information


Supporting File 1

Supporting File 2

## Data Availability

The data presented in this study are available on request from the corresponding authors.
