# Supplementary material for: Jing Si Herbal Tea Aqueous Extract Modulates G‐CSF‐Induced Hematopoietic Stem and Progenitor Cells Mobilization and Mitigates Associated Pain in a Murine Model
Source: Cell Biochem Funct. 2026 Nov 2;44(11):e70335. doi: 10.1002/cbf.70335 (PMC13725352; doi:10.1002/cbf.70335)
Supplement: Supplementary file 1 [file CBF-44-e70335-s001.docx]

# Supplementary Material

# HPLC/MS results

# Instrument Device :

| **Instrument Categories** | **Instrument Model** |
| --- | --- |
| Pump Model | Agilent 1260 G1312B Binary Pump |
| AutoSampler | Agilent 1260 G1367E HiP ALS Autosampler |
| Degasser | Agilent 1260 G1379B Degasser |
| Column | Phenomenex Kinetex-Phenyl-Hexyl-100A (100 mm x 2.1 mm i.d., 2.6 um) |
| Mass Spectrometer | AB Sciex Instruments QTRAP 5500 |
| Source type | Turbo V Ion Source |

**HPLC Method Properties :**

Duration	: 25 min Injection volume	: 5 ul

Mobile Phase	: A: 0.1% ( v/v ) Formic acid / Water ; B: 0.1% ( v/v ) Formic acid / Acetonitrile

Step Table	:

| Step | Total Time(min) | Flow Rate(μl/min) | A (%) | B (%) |
| --- | --- | --- | --- | --- |
| 0 | 0.10 | 120 | 95.0 | 5.00 |
| 1 | 5.00 | 120 | 95.0 | 5.00 |
| 2 | 8.00 | 120 | 50.0 | 50.0 |
| 3 | 10.0 | 120 | 5.00 | 95.0 |
| 4 | 15.0 | 120 | 5.00 | 95.0 |
| 5 | 16.0 | 120 | 95.0 | 5.00 |
| 6 | 25.0 | 120 | 95.0 | 5.00 |

# Sample Preparation :

An aliquot of 30 µL of the sample was mixed with 90 µL of methanol. The mixture was vortexed thoroughly to ensure complete dissolution. It was then incubated at -20 °C for 30 minutes. After incubation, the sample was centrifuged at 15,000 × g for 10 minutes. The resulting supernatant was carefully collected and diluted 2-fold with 50% methanol prior to analysis. The overall dilution factor applied was 8×.

# Mass Spectrometer Information :

Scan Type	: MRM (Multiple Reaction Monitor)

Polarity	: Positive ion mode

Source temperature	: 500 ℃

Data acquisition	: Analyst 1.5 software

# Mass spectrometer parameter setting :

CUR (curtain gas)	: 20.00 psi Nebulizing gas ( GS1 ) : 40.00 psi Collision-activated Dissociation (CAD) : High	Heating gas ( GS2 ) : 50.00 psi Electrospray capillary voltage	: 5000.00 V

# Preparation of Calibrators :

All standard samples were initially dissolved in solvent to obtain solutions at various concentrations. A mixed working solution was then prepared by combining the standards and diluting them with 50% methanol to achieve concentrations in the microgram per milliliter (µg/mL) range. This working solution was stored at -20 °C for subsequent use. Calibration solutions were freshly prepared prior to analysis by serial dilution of the working solution with 50% methanol, resulting in a concentration range spanning from µg/mL to ng/mL.

# Quantitation Method :

The Multiple Point External Standard method was employed for quantitation.

# Selected Transitions and Parameter Settings

| Analytes | Q1 mass | Q3 mass | DP (V) | EP (V) | CE (V) | CXP (V) |
| --- | --- | --- | --- | --- | --- | --- |
| Harmine | 213.2 | 144.2  170.2  198.2 | 220 | 10 | 56  43  31 | 16  13  19 |
| Swertisin | 447.2 | 297.2  381.2  429.2 | 90 | 10 | 34  22  18 | 50  26  35 |
| Isoliquiritigenin | 257.2 | 119.2  137.2  147.2 | 25 | 10 | 38  35  26 | 13  10  13 |
| Eupatilin | 345.2 | 169.2  315.3  330.2 | 120 | 10 | 50  43  34 | 13  22  28 |
| 3’,4’-Dimethoxyflavone | 283.2 | 222.2  239.2  267.2 | 130 | 10 | 34  37  35 | 20  22  22 |
| Nerolidol | 205.2 | 109.2  121.2  149.2 | 30 | 10 | 20  19  14 | 15  22  11 |

**Note:** Red indicates the ion pairs used for quantitation.

Since DMF and Nerolidol were only be detected in the positive mode, in order to analyze all compounds

in the same Run, the data in the positive mode were used**.**

# Calibration Curve of Standard

**Linear regression	Weighting**：**1/x**

Note:

1. Since the signal sensitivity of Nerolidol was not high, it was easily covered by other highly sensitive signals.

2. From the chromatogram, it could be seen that the selected ion pairs could all show signals at the same RT time, indicating their specificity.

3. Since the actual sample composition was complex and the concentration of each compound was different, it was difficult to present all of them in one spectrum; they could only be presented separately in their respective XIC graphs, as shown below:

**Sample Spectrum**

Jing Si Herbal Tea

**Analyte Name:**	Harmine

Regression Equation:	y = 1.39e+005 x + 7.88e+004 (r = 0.9998)

| **Expected Concentration (ng/ml)** | **Mean**  **Calculated Concentration (ng/ml)** | **% Accuracy** |
| --- | --- | --- |
| 1 | 0.87 | 86.8 |
| 2 | 1.89 | 94.7 |
| 4 | 4.20 | 105.0 |
| 10 | 10.44 | 104.4 |
| 20 | 20.88 | 104.4 |
| 40 | 41.03 | 102.6 |
| 100 | 104.17 | 104.2 |
| 200 | 201.17 | 100.6 |
| 400 | 388.08 | 97.0 |
| 1000 | 1004.28 | 100.4 |

**Analyte Name:**	Swertisin

Regression Equation:	y = 3.7e+004 x + 3.83e+003 (r = 0.9997)

| **Expected Concentration (ng/ml)** | **Mean**  **Calculated Concentration (ng/ml)** | **% Accuracy** |
| --- | --- | --- |
| 0.223 | 0.18 | 82.3 |
| 0.446 | 0.43 | 96.0 |
| 0.892 | 0.89 | 99.7 |
| 2.23 | 2.27 | 101.9 |
| 4.46 | 4.62 | 103.6 |
| 8.92 | 9.21 | 103.2 |
| 22.3 | 23.07 | 103.4 |
| 44.6 | 45.91 | 102.9 |
| 89.2 | 93.72 | 105.1 |
| 223 | 233.76 | 104.8 |
| 446 | 436.72 | 97.9 |
| 892 | 883.50 | 99.0 |

**Analyte Name:**	Isoliquiritigenin

Regression Equation:	y = 1.79e+005 x + 3.91e+004 (r = 0.9995)

| **Expected Concentration (ng/ml)** | **Mean**  **Calculated Concentration (ng/ml)** | **% Accuracy** |
| --- | --- | --- |
| 0.05 | 0.04 | 83.0 |
| 0.1 | 0.10 | 98.5 |
| 0.2 | 0.20 | 98.0 |
| 0.5 | 0.50 | 99.0 |
| 1 | 1.05 | 105.5 |
| 2 | 2.06 | 102.9 |
| 5 | 5.11 | 102.3 |
| 10 | 10.32 | 103.2 |
| 20 | 20.74 | 103.7 |
| 50 | 53.27 | 106.5 |
| 100 | 99.63 | 99.6 |
| 200 | 195.84 | 97.9 |

**Analyte Name:**	Eupatilin

Regression Equation:	y = 5.19e+005 x + 4.34e+004 (r = 0.9998)

| **Expected Concentration (ng/ml)** | **Mean**  **Calculated Concentration (ng/ml)** | **% Accuracy** |
| --- | --- | --- |
| 0.17215 | 0.14 | 80.7 |
| 0.3443 | 0.33 | 95.3 |
| 0.6886 | 0.71 | 103.3 |
| 1.7215 | 1.79 | 104.1 |
| 3.443 | 3.67 | 106.7 |
| 6.886 | 7.17 | 104.1 |
| 17.215 | 18.12 | 105.3 |
| 34.43 | 35.40 | 102.8 |
| 68.86 | 67.84 | 98.5 |
| 172.15 | 170.73 | 99.2 |

**Analyte Name:**	DMF

Regression Equation:	y = 6.06e+005 x + 7.36e+004 (r = 0.9997)

| **Expected Concentration (ng/ml)** | **Mean**  **Calculated Concentration (ng/ml)** | **% Accuracy** |
| --- | --- | --- |
| 0.14115 | 0.12 | 82.7 |
| 0.2823 | 0.26 | 91.7 |
| 0.5646 | 0.58 | 101.9 |
| 1.4115 | 1.49 | 105.3 |
| 2.823 | 2.99 | 106.0 |
| 5.646 | 5.90 | 104.6 |
| 14.115 | 14.99 | 106.2 |
| 28.23 | 29.29 | 103.7 |
| 56.46 | 56.10 | 99.4 |
| 141.15 | 139.11 | 98.6 |

**Analyte Name:**	Nerolidol

Regression Equation:	y = 8.07e+003 x + 2.29e+004 (r = 1.0000)

| **Expected Concentration (ng/ml)** | **Mean**  **Calculated Concentration (ng/ml)** | **% Accuracy** |
| --- | --- | --- |
| 0.5 | 0.42 | 83.1 |
| 1 | 1.00 | 100.5 |
| 2 | 2.13 | 106.5 |
| 5 | 5.06 | 101.2 |
| 10 | 10.01 | 100.1 |
| 20 | 20.67 | 103.4 |
| 50 | 52.16 | 104.3 |
| 100 | 100.42 | 100.4 |
| 200 | 201.43 | 100.7 |
| 500 | 502.07 | 100.4 |
| 1000 | 996.89 | 99.7 |
| 2000 | 1996.24 | 99.8 |

Harmine

| **Sample Name** | **Spectrum** | **Calculated concentration ( ng/ml )** |
| --- | --- | --- |
| **Jing Si Herbal Tea** |  | **N.C.** |

Swertisin

| **Sample Name** | **Spectrum** | **Calculated concentration ( ng/ml )** |
| --- | --- | --- |
| **Jing Si Herbal Tea** |  | **2510.0** |

Isoliquiritigenin

| **Sample Name** | **Spectrum** | **Calculated concentration ( ng/ml )** |
| --- | --- | --- |
| **Jing Si Herbal Tea** |  | **179.00** |

Eupatilin

| **Sample Name** | **Spectrum** | **Calculated concentration ( ng/ml )** |
| --- | --- | --- |
| **Jing Si Herbal Tea** |  | **279.00** |

3’,4’-Dimethoxyflavone

| **Sample Name** | **Spectrum** | **Calculated concentration ( ng/ml )** |
| --- | --- | --- |
| **Jing Si Herbal Tea** |  | **0.0217** |

Nerolidol

| **Sample Name** | **Spectrum** | **Calculated concentration ( ng/ml )** |
| --- | --- | --- |
| **Jing Si Herbal Tea** |  | **15.400** |

# Summary (The precise concentration of each metabolite in the sample; unit : ug/ml)

**Calculation Formula:** Analyte calculated concentration x 8 (dilution factor)

| Analyte Name | Harmine | Swertisin | Isoliquiritigenin | Eupatilin | 3’,4’-  Dimethoxyflavone | Nerolidol |
| --- | --- | --- | --- | --- | --- | --- |
| **Jing Si Herbal Tea** | **N.C.** | **20080** | **1432.0** | **2232.0** | **0.1736** | **123.20** |

Note:

1. Due to the different properties of different metabolites, we could not guarantee that the processing steps taken were the best methods; if the reader had doubts about its efficiency, he/she could specify the preparation method and we will try our best to cooperate.
2. N.C. => Not Calculated. Peaks were detected, but their integrated area was far below the minimum quantitative limit, so the software could not convert the concentration.
3. In this analysis, the minimum calibration concentration had been diluted down by ~10 times. There were still many samples that could measure the peak, but the concentration could not be converted, indicating that the content of these components was quite low.
4. The concentrations of certain analytes clearly exceeded the linear range of detection. However, further dilution would likely result in the loss of detectable signals for other analytes. Therefore, an 8-fold dilution was adopted as a compromise. Please take note of this limitation.
