# Supplementary material for: Jing Si Herbal Tea Aqueous Extract Modulates G‐CSF‐Induced Hematopoietic Stem and Progenitor Cells Mobilization and Mitigates Associated Pain in a Murine Model
Source: Cell Biochem Funct. 2026 Nov 2;44(11):e70335. doi: 10.1002/cbf.70335 (PMC13725352; doi:10.1002/cbf.70335)
Supplement: Supplementary file 2 [file CBF-44-e70335-s002.docx]

**Supplementary Figures**

**Supplementary Figure 1.**

**JSHT reduces the inflammatory factor TNFα produced by microglia induced by G-CSF in the spinal cord.**

Tissue section images were captured individually using an OLYMPUS® BX53 microscope (Olympus® Corporation, Tokyo, Japan) equipped with a fluorescence imaging system. Magnification: 40X.
